# Reconfiguring Vulnerability and Dis/Ability: An Agential Realist Exploration to Disentangle Vulnerability Effects in Austria's COVID‐19 Response

**DOI:** 10.1111/1467-9566.70035

**Published:** 2025-04-07

**Authors:** Oliver Koenig, Sabine Mandl, Simon Reisenbauer

**Affiliations:** ^1^ Bertha von Suttner Private University St. Pölten Austria

**Keywords:** activist affordances, COVID‐19, crisis response, debilitation, entanglement, response‐ability, vulnerabilities, vulnerability effects

## Abstract

This article examines how vulnerability emerged, evolved and was contested during Austria's COVID‐19 response, by attending to the entangled realities of people with dis/abilities. Using a posthumanist, agential realist lens and a diffractive methodology, the research explores how vulnerability is not a fixed state but a dynamic process shaped by material and discursive practices. It introduces the concept of ‘vulnerability effects’ to articulate that vulnerabilities are simultaneously a product of and a catalyst for material and discursive practices within systems of dis/ability and crisis response. Drawing on Carol Thomas’s distinction between disablism and impairment effects, the analysis moves beyond binary framings to capture how vulnerabilities are simultaneously produced by systemic inequalities and contested through creative acts of resistance. Through the narratives of participants navigating institutional restrictions, inaccessible environments and intersecting crises, the article illustrates how debilitating conditions and activist affordances intra‐act, shaping the possibilities for agency and resilience. The findings reveal the fluid, context‐dependent and performative nature of vulnerability, challenging static paradigms in crisis response. By reframing vulnerability as relational and emergent, the article calls for inclusive and response‐able approaches to policy and social structures that address systemic neglect and promote equitable opportunities.

## 1 Introduction

The COVID‐19 pandemic altered daily life for individuals worldwide (OHCHR 2020). As public health measures were dismantled, their visible traces—such as empty disinfection dispensers—became reminders of a crisis consigned to history. This retreat into collective amnesia, evident in social, political and media discourses, undermines the opportunity to critically engage with the structural inequalities and systemic neglect that the pandemic exposed.

Our research documents and analyses the impact of COVID‐19 on people with dis/abilities^1^ and complex mental health conditions. *Cov_enable*—a collaborative project realised by *The Bertha von Suttner Private University and the University of Vienna* from May 2021 to April 2025—employs a participatory, qualitative and longitudinal approach to observe how conceptions of vulnerability are reshaped and travel between the macrolevel forces of neoliberal capitalist structures that shape societal understandings of dis/ability, the mesolevel institutional practices that mediate access to self‐determined supports and the microlevel lifeworlds in which individuals adapt, resist or innovate.

In this article, we aim to critically examine the evolving nature of vulnerability during the pandemic through a posthumanist lens, inspired by Barad's (2007) agential realism. We will do so by introducing the emergent narrative of *vulnerability effects*, a conceptual framework that captures how vulnerabilities are simultaneously a product of and a catalyst for material and discursive practices within systems of dis/ability and crisis response. By adopting vulnerability as an analytical lens, we trace *not what vulnerability is but what vulnerability does and how it comes into being*.

By engaging with three narratives^2^ of research participants, we highlight the relational and fluid nature of vulnerability. Through this lens, we aim to challenge dominant crisis paradigms, calling for response‐able approaches that are both just and transformative (Tronto 2023) while deepening theoretical engagements within critical posthumanist disability studies (Goodley et al. 2021) and offering insights to reimagine inclusive crisis responses.

This article opens by contextualising Austria's fluctuating crisis responses. Although the COVID‐19 pandemic is often framed as a moment of exceptionality, such a view risks obscuring the structural conditions and historical patterns that have long influenced constructions and responses to vulnerability. Hence, we consider vulnerability as a travelling concept, reflecting its evolving meanings during the pandemic within broader sociohistorical contexts.

We then introduce a posthumanist, agential realist perspective, focusing on how the concepts of entanglements and intra‐activity enrich our understanding of vulnerability. Next, we detail the diffractive research methodology applied in this study.

At the heart of the article, we reread three participant narratives, each offering a perspective on the variety of institutional contexts, material conditions and personal activism shaping the lives of adults with dis/abilities. Finally, we outline the emergent narrative of *vulnerability effects* for understanding vulnerability as a dynamic interplay between material and discursive practices. Our analysis disentangles the ways in which these dimensions co‐constitute, sustain and transform the experience of vulnerability in crisis contexts.

## 2 Fluctuating Solidarities and Institutionalised Neglect: Austria’s Pandemic Response

In Austria, the government’s initial response to COVID‐19 identified ‘vulnerable groups’—including individuals with pre‐existing health conditions, impairments, mental illnesses and older adults (BMSGPK 2020)—as a primary focus of protective measures. However, this categorisation resulted in significant disparities in the implementation of restrictions. Although the general population experienced mobility constraints for only a few weeks, individuals in institutional care endured prolonged confinement, including months‐long visitation bans and curfews (Volksanwaltschaft 2020; Koenig and Barberi 2023).

Simultaneously, essential COVID‐19 protective measures—such as tests, masks and personal protective equipment—were frequently inaccessible to persons with dis/abilities living independently. Only through the concerted efforts of members of the independent living movement were the particular needs of persons supported by personal assistance schemes recognised and subsequently considered in the implementation of emergency measures and supply strategies (Monitoringausschuss 2021).

The initial phase of the pandemic in Austria was dominated by a discourse of fear, exemplified by former Chancellor Sebastian Kurz's assertion: ‘We will soon face the situation in Austria where everyone knows someone who has died from Corona’ (Czejkowska and Froebus 2021, 96). This narrative served to justify restrictive measures by invoking collective solidarity and responsibility. However, as the pandemic progressed, the political emphasis shifted from the protection of ‘vulnerable groups’ to the broader goal of preserving population‐wide healthcare systems.

Austria became the first European country to implement a national COVID‐19 vaccine mandate. Announced in November 2021, the mandate required vaccination for everyone over the age of 18, with penalties for noncompliance (Druml and Czech 2022). This policy marked a historic shift and provoked significant social opposition as debates over the mandate became increasingly polarised.

By summer 2022, Austria had significantly relaxed its protective measures, abolishing the vaccine mandate and gradually lifting all mask‐wearing requirements (Parliament, 7 July 2022). Those identified as ‘worthy of protection’ were increasingly left without any safeguards and accommodations.

Austria’s pandemic response exemplifies a fluctuating focus on dis/abled individuals, oscillating between moments of recognition and extended periods of neglect (Naue and Flieger 2022). Political and institutional attention disproportionately centred on individuals in institutional care settings, overlooking independent living arrangements. Stereotypical portrayals of people with dis/abilities as dependent and pitiable in both political and media discourses during COVID‐19 (de Ruiter, Dekking et al. 2023) contributed to the reinforcement of paternalistic practices and the legitimisation of restrictive institutional measures.

In the midst of these dynamics, vulnerability emerges as a relational and ongoing process shaped by inequalities, material reality and discourse. The next chapter explores these intersections, situating the pandemic within a continuum of historical and social processes.

## 3 The Mutable Terrains of Vulnerability

Vulnerability, as a concept, has travelled through different terrains of understanding and application, shaped by historically contingent and socially embedded conditions. In its earliest usages, vulnerability was tied to individual susceptibility to harm, particularly in medical or military contexts where the focus lay on securing the body against threats (ten Have 2016). During the 1980s and 1990s, vulnerability began to be framed within broader structural and social dimensions. Responses to HIV/AIDS and environmental crises revealed how societal inequalities, stigma and exclusion intersected with individual experiences of harm (Higgins et al. 2010; Brown 2011). These shifts reflected an increasing recognition that vulnerability is not merely an inherent condition of certain individuals but a socially and politically mediated phenomenon.

The COVID‐19 crisis yielded a research field where vulnerability concepts have been applied in various manners. This often entailed an essentialising focus on individual medical vulnerability, with less emphasis on the exploration of structural dimensions (Bambra et al. 2021; Phillips 2021; The Lancet 2020).

The binary framing of vulnerability as individual fragility or a product of precarious systemic inequalities often rests on the dichotomy of ‘capacity‐endowed’ and ‘debility‐laden bodies’ (Puar 2009, 169). Puar's (2020) work disrupts this binary by introducing debility, thus shifting the focus from the capacities of dis/abled bodies to the historically contingent and politically charged systems that produce and sustain able‐bodiedness. Debilitation is not a static condition but a process embedded in the relational assemblages of legal frameworks, infrastructural adaptations and sociopolitical norms. So too, capacitation emerges as a regulated and extractive relation. This perspective exposes how it is often systemic neglect, rather than overt violence, that operates as a mechanism of governance, actively producing vulnerability.

During the pandemic, these dynamics were especially evident in institutional care settings where confinement and isolation were implemented under the guise of protection (de Ruiter, Niemeijer, et al. 2023). The Disability Covid Chronicles project (Mills et al. 2025), a 3‐year research project and collated online archive, provides insights into these intersections, documenting experiences of amplified risks and systemic neglect among dis/abled individuals. Among this assemblage, Michele Sommerstein's blog (Sommerstein, n.d.) provides reflections on the inaccessibility of healthcare resources and structural discrimination that contributed to disproportionate outcomes. Yet, these accounts showcase not only the harms faced by dis/abled individuals but also highlight their creative responses, mutual aid and activism networks. Initiatives such as the ‘Mask Up’ campaign, where activists distributed protective gear at public transit hubs, embody the collective efforts to address the systemic neglect dis/abled communities faced.

Dokumacı (2023) offers a lens to understand these acts of resistance through the concept of activist affordances—the microperformances by which dis/abled individuals navigate and transform hostile environments. These ‘tiny everyday artful battles’ (6) reconfigure spaces of exclusion into opportunities for more liveable worlds. Through such engagements, dis/abled individuals craft unique worlds of expressivity and legibility, not as a result of systemic support but as acts of accommodation arising despite systemic impediments.

As Tronto (2023) argues, responses to crises such as COVID‐19 are deeply entangled with ethical and political considerations. Vulnerability arises in the spaces where structural inequalities, epistemic injustices and material conditions intersect (Mladenov and Dimitrova 2023), shaping both the possibilities for agency and the constraints imposed by societal systems.

The COVID‐19 pandemic, far from an isolated crisis, serves as a lens through which to examine vulnerability as a relational and performative concept. Posthumanism and agential realism offer a framework for further exploring these material‐discursive entanglements, emphasising intra‐action as central to understanding the systemic workings of dis/ability and crisis response.

## 4 Posthumanism and Agential Realism

Posthumanism offers a transformative perspective for rethinking the concept of vulnerability by challenging anthropocentric assumptions that have historically shaped academic and policy discourses. Emerging in the early 1990s, posthumanism has evolved into an intellectual framework for understanding the relational nature of material and social realities (Herbrechter et al. 2022). It departs from traditional humanist paradigms by rejecting the notion of humans as distinct and superior entities, advocating instead for a relational ontology that recognises the interdependence of human and more‐than‐human agencies (Braidotti 2019).

Within critical disability studies, posthumanism has become an important lens for interrogating the interplay of technological, ecological and social forces that impact human bodies. Goodley et al. (2021) argue that posthumanism allows for a nuanced examination of the interplay between human beings, who are increasingly empowered through technological advancements yet simultaneously more vulnerable due to ecological impacts. They suggest that posthumanism in disability studies not only questions traditional dichotomies—such as those between humans and machines—but also promotes a more constructive and imaginative engagement with these entities (Goodley et al. 2021, 31).

Karen Barad’s agential realism, influenced by Niels Bohr’s philosophy of quantum mechanics, deepens this perspective by offering the premise that the epistemological, the ontological and the ethical are intertwined, making it impossible to separate them. Central to agential realism are the concepts of intra‐action and entanglement. With her concept of intra‐action, Barad (2007) reassesses the role of matter as dynamic and variable, rather than static and unchanging. Agency is not an inherent property of subjects or objects but is understood as the ‘mutual constitution of entangled agencies’ (33). This redefinition contrasts with the concept of interaction, where entities are seen as pre‐existing independently. Instead, in an intra‐active framework, entities emerge and continually materialise through their entanglements with humans, nonhumans or the environment (Barad 2007). This shift encourages a view of the world as inherently composed of interdependent phenomena.

Barad's notion of entanglement further redefines the material‐discursive relationship, rejecting the binary separation of the material (nonhuman, reality) and the discursive (human, social). Instead, entanglements reflect irreducible relations of responsibility and obligation, where the boundaries of ‘self’ and ‘other’ are continually reconfigured through intra‐action (Barad 2014).

Vulnerability is not an inherent or static condition but results from dynamic intra‐actions shaped by systems of power and knowledge. Barad (2007) emphasises boundary‐making practices ‘formative of matter and meaning, productive of and part of the phenomena produced’ (148). These *apparatuses* delineate inclusion and exclusion, defining what matters within particular intra‐actions. The COVID‐19 pandemic has highlighted how vulnerability is materialised through these processes, revealing ‘able‐bodiedness’ not as a natural state but as a specific embodiment ‘co‐constituted through the boundary‐making practices that distinguish “able‐bodied” from “disabled”’ (Barad 2007, 158).

Posthumanism and agential realism shift the focus from individual characteristics to systemic forces, positioning vulnerability as a dynamic phenomenon shaped by entangled intra‐actions and boundary‐making practices. They highlight the sociopolitical and socioeconomic conditions that produce vulnerability and urge a critical rethinking of inclusion and exclusion as co‐constituted and relational.

## 5 Tracing Vulnerability Through Diffractive Methodologies: Mapping Entanglements in Crisis Research

Our research and methodology did not follow a linear progression. Instead, it unfolded within the entangled realities of conducting research under the conditions of COVID‐19, demanding that we adapt both our conceptual frameworks and practical research approaches to the evolving material and social realities (Nind et al. 2023). The pandemic shaped not just the subject of our research—vulnerability—but also the processes of knowledge production. For example, the first series of interviews had to be planned as remote conversations, mediated by unstable digital infrastructures and shaped by participants' varying levels of access and comfort with technology. Producing diary entries—a research strategy we adopted—was similarly shaped by material conditions, such as the availability of quiet spaces during lockdowns, the emotional weight of isolation and the affordances of the devices provided. In our project, we adopted a two‐stream research approach to capture both structural dynamics and individual experiences of vulnerability.

The first stream aimed to uncover emerging discourses surrounding vulnerability and the material practices evolving within institutional responses. We monitored policies linked to COVID‐19 and analysed media representations through datasets from national television (public broadcasting) and newspapers. In parallel, we conducted interviews and group discussions with stakeholders, including leaders of crisis management boards and representatives of organisations.

The second stream focused on the situated and relational dynamics shaping the lives of people with dis/abilities and mental illness. The sampling rationale was informed by theoretical sampling principles that allowed for both narrow and broad comparisons, spanning opposite polarities on the inclusion–exclusion divide reflecting the ideological and structural diversity of care and support arrangements. Specifically, we selected research participants from the following:

- Traditional group‐home‐based models of care catering predominantly to individuals with intellectual dis/abilities.
- Decentralised community‐based care providers focusing on services for individuals with mental health needs.
- Dis/ability‐led providers offering personal assistance to individuals with physical impairments residing in their own homes.

Our project adhered to rigorous ethical standards; however, due to its nonclinical nature, no formal ethical approval was required through its funding agency. Nonetheless, the project implemented a multistage informed consent process to accommodate the diverse needs of participants, including accessible communication formats and options for partial or complete withdrawal of consent at any stage of the research. In framing our ethical approach, we distinguished between ethical requirements and ethical responsibilities (Anderson and Muñoz Proto 2016). Ethical requirements included adherence to the European General Data Protection Regulation (GDPR), encompassing measures to ensure data confidentiality and security. Ethical responsibilities reflected the project’s epistemological and normative commitments to participatory research. Recognising the potential for exclusion and marginalisation in research processes, the project prioritised principles of voice, empowerment and self‐representation to ensure that participants could meaningfully shape their engagement.

Data generation began with semistructured interviews (Gibson et al. 2013) in mid‐2021. Participants who opted in engaged in personal diary entries (Bates 2020) for a period spanning from 6 months to a year. These participants received personal tablets for submitting their entries, giving them a choice on whether to produce their diaries as videos, audios or written texts. Follow‐up interviews were scheduled based on preliminary analyses, which were then discussed and refined in collaboration with the participants. In total, we conducted 60 interviews involving 34 individuals. Among them, 12 participants submitted about 100 diary entries.

Engaged in diligent attempts to code, categorise and draw out lines of connection between the data, we frequently encountered what felt as *dead ends*. The ‘apparatus of knowing’ (Barad 2007) we had established seemed to restrict our understanding and ability to capture the dynamism unfolding in our engagements with research participants. A sense of frustration permeated our team as it felt that the practices of knowing we were employing only mirrored back to us our pre‐existing understandings of vulnerability (Mazzei 2014).

It was rather by chance that we stumbled upon a text about posthumanist diffractive methodologies for dis/ability studies (Naraian and Gabel 2023). In this text, Naraian and Gabel (2023) engage with an experience‐driven engagement of postqualitative research methodologies and discuss these methodologies for their ability to engage with the complexities of disabled experiences and explore the underlying power dynamics and material conditions.

Central to the reshaping of our methodical approach became the concept of *diffraction*. Originating from an optical phenomenon, this term has been adopted by Haraway (1997) and Barad (2014) to signify a methodological shift in knowledge practices. Diffraction, in its physical sense, refers to the meeting and deflection of waves by an obstacle, leading to interference and the creation of new patterns. In contrast to reflection, which reproduces mirroring or sameness, a diffractive approach to analysis implies not viewing difference as either an inherent essence or something inconsequential but as relational and dynamic. As Haraway (Haraway 1999, 320) notes, “a diffraction pattern does not map where differences appear but rather maps where the *effects* of differences appear.” Barad (2007, 72) builds on this, refining diffraction as an analytical tool for understanding and responding to the *effects of difference*.

Moving on from our initial coding and categorisation, we began to incorporate Clarke’s (2005) situational analysis, particularly the posthumanist elements elaborated in later versions (Clarke et al. 2018, 2022). By incorporating Barad’s concepts of entanglement and intra‐action (Barad 2007), we embraced the possibilities of diffractive analysis to explore vulnerability not as interactions of separate entities but as entanglements of diverse intra‐actional agentic forces (Schwertel 2023). Just as vulnerability must be understood as a *concept in motion*, the notion of travelling concepts, experiences and sense‐making highlights the fluidity and situatedness of knowledge—an essential principle in situational analysis (Clarke et al. 2018, 42) and diffractive methodology (Fox and Alldred 2023).

**FIGURE 1:**
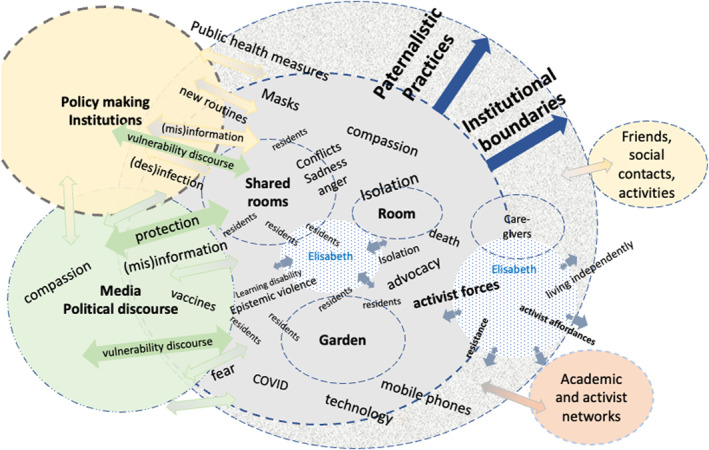
Diffractive mapping Elisabeth, working version 27.04.2024.

Mapping, both as a physical and cognitive tool, played a critical role in our analysis. Early working versions foregrounded participants’ embeddedness in institutions, discourses and practices. As the research team deepened its engagement with diffractive analysis, the maps became more fluid, illustrating the cascading effects of policy changes on participants' daily lives and showing how intra‐acting elements materialised differently across diverse sociomaterial contexts (Figure 1).

Flip charts and other tools were used to work with these maps during analysis sessions. More than representational tools, these evolving maps functioned as analytic devices, making visible shifting entanglements, tensions and the recursive effects of systemic responses.

Although the pandemic created changes in the lived material realities of people globally, within the lives of people with dis/abilities whom we accompanied during the research, we began to notice the workings of a distinct layer of *difference within the difference* of the pandemic, which we have coined as *vulnerability effects*.

Doing diffractive analysis meant reading and rereading our data and rearranging our maps through and with (multiple) theoretical concepts, especially material‐discursive entanglements and intra‐action (Barad 2007), activist affordances (Dokumacı 2023) and debilitation (Puar 2020). This allowed us to trace how the effects of policies (e.g., lockdown measures) were unevenly materialised in participants’ lives.

The following diffractive readings are an attempt to generate meaning by capturing the complex realities and experiences of our research participants. The selection of the three reread narratives presented here reflects the purposive sampling strategy established at the outset of the project, each of which highlights the relational interplay of structural, material and discursive forces that continually shape the living and support conditions of adults with dis/abilities.

## 6 Trying to Stay Sane

John, a man in his thirties, identifies as a person living with a psychiatric impairment. Across four interviews and seven audio diaries between June 2021 and July 2022, he shared how his life evolved amid the pandemic’s disruptions. John lives in a semi‐supported living arrangement, supplemented by family support, and spends his days at a sheltered workshop. After the workshop, he heads back home to his apartment where he interacts with his family, walks his dog and engages in recreational engagements, such as video gaming.

John's story was selected for this analysis because it reveals the complex relational entanglements of various forces, including social context, psychiatric symptoms and the social and material infrastructure shaping his evolving mental health within community‐based psychiatric support systems.

The onset of the pandemic altered the fabric of John's social and material world. He describes an initial sense of heightened social empathy and respect for personal space in public encounters, counterbalanced by the burden of adapting to shifting regulations. Central to his narrative is his management of psychiatric symptoms:

The hallucinations inflict pain on me. […] They are always present, even at this moment, they are *always* present. At the moment they’re very quiet (.) they just sit there and watch us, it’s intense, yes.

(Interview, 13 September 2021)

For John, these hallucinations are as tangible as a table is for someone else, making them a constant material agent in his life. His self‐management routines, centred on carefully structured rituals, provide predictability amid these disruptions. Medications, for example, act as temporal anchors for his day, organising his activities and framing his sense of control:

So, a typical day. I get up at six thirty, take my stomach acid tablet, because after all I have nine medications in total, then I have breakfast, take my medication, the standard medication […] And then I take my evening medication, go to sleep, get everything ready for the next day, so that I only have to get up, get dressed, take my medication, eat and leave.

(Interview, 13 September 2021)

The lockdowns amplified John’s reliance on his family, with their proximity becoming both a crucial safety net and a source of confinement. Over time, he felt increasingly restricted by this dependence, spurring aspirations for greater independence and prompting him to consider moving to a new apartment. He began learning household tasks:

Now, thank God, I can already carry my laundry down to the cellar, then into the washing machine, the detergent, the fabric softener and then switch it on. My mother was so kind […] to mark exactly what I need for my laundry with a smiley and now I know, ok, turn the smiley, press start and then my laundry will be washed.

(Interview, 13 September 2021)

These practical adaptations reflect a broader sociomaterial engagement where John’s personal agency, the materiality of his environment and the emotional and practical support of his family collectively contribute to his evolving sense of self‐sufficiency.

At the same time, the pandemic led to drastic shifts in his social context. Previously an active member of a card gaming community, John’s role as a convener was disrupted. Although adapting to digital communication technologies allowed for some continuity, it left him physically isolated and intensified his psychiatric symptoms. He describes the challenge of living alone:

Living alone and being mentally ill, I mean, I mean, there are challenges that everyone has when they live alone, but mental illness makes it even more so that you have to be careful, yes? What was difficult for me was being alone with myself. That wasn’t the case before. It’s a challenge, but it’s part of it. Because how can you become independent if you can’t even be alone with yourself? With thoughts that keep going round and round and these future scenarios that you just kind of run through.

(Audio diary, 30 January 2022)

An important agentic force—operating both materially and discursively—in the entanglements of his mental states is the societal response to shifting rules and regulations and the onset of overlapping crises, mediated and amplified through media. His foundational anxiety, which he refers to as ‘Grundangst’, heightened by these conditions, results in the layering of multiple dimensions of anxiety:

I have to admit that I sit at home more often now and really have to prevent my thoughts from slipping into extremes […] It’s corona‐related, yes. I mean, I used to get caught up in things and completely overthink them, in these “what ifs”, “what could have been”? All these factors that you can’t really influence as an individual anyway. And that’s becoming more and more, and the fear that I’m now getting, or already have of corona, is becoming an increasingly intense experience.

(Interview, 4 February 2022)

John’s anxiety, amplified by a cascade of ‘what if’ scenarios and the effects of a constant intensification of stress as a result of witnessing growing public disregard for safety measures and conspiracy theories affecting his peers, as well as global events such as Russia’s invasion of Ukraine, culminated in a mental health crisis early in 2022, which necessitated a period of hospitalisation disrupting his plans for greater independence. In response, the collective advice from his support network—including his family, doctor and support worker—was to postpone his transition towards living more independently.

To navigate this new situation, John relied on his professional support networks. The consistent involvement of his doctor and support worker, sustained through regular phone calls, proved crucial in his care. As a key strategy to maintain mental stability, John talks about employing behavioural therapy techniques he describes as ‘tics’. These tics are double‐edged. On one hand, they provide a sense of order, affording him a sense of stability during moments of distress. On the other hand, they are constant reminders of the need for vigilance, imposing a mental burden that can be exhausting:

My brain would be completely overwhelmed with sensory impressions because of the sounds of other people, all the images that I perceive and such. And then I usually listen to loud music or read in a book. […] That was before COVID that I needed music but because of COVID I've now moved on to very loud music, like hardcore techno music, very, very loud heavy metal.

(Interview, 13 September 2021)

Spending time outdoors introduces another paradox: it offers a sense of spatial liberation and social validation yet simultaneously amplifies his anxieties. Regulating the volume of the music he listens to becomes a crafting device to shield him from the sensory overload of public spaces and to prevent the escalation of his symptoms. The pandemic intensified John’s hallucinations, necessitating his coping mechanisms or ‘tics’, to become more potent. His strategy of ‘freezing like a stone’ in crowded settings (audio diary, 13 January 2022) emerges as a last‐resort mechanism.

John's anxieties and responses are not static but continually intra‐act with the dynamic interplay of restrictions and freedoms. The persistent nature of his symptoms, exacerbated by the pandemic’s shifting conditions, begins to debilitate the agency he strives to maintain. A speculated longer period of hospitalisation in a psychiatric facility in the summer of 2022 indicates a peak in this layered emotional journey.

## 7 Staying in Control Amid Inaccessible Environments

Sophia, an entrepreneur in her 50s, has spent over 2 decades managing an animal farm where she hosts guests and organises events. Sophia is blind and uses personal assistance, which is essential for both her professional activities and personal mobility. She lives with and cares for her frail mother. Her narrative consists of an interview conducted in July 2021 and a series of 20 diary entries covering the period until June 2022.

Sophia’s narrative was chosen because her experiences crystallise how certain differences come to matter distinctly in the lives of people with dis/abilities and how the trajectory of her life is re‐steered by the interplays of various entangled agential forces—the inaccessibility of both physical spaces and sociotechnical environments, coupled with policy biases and persistent negative attitudes towards dis/ability—intra‐acting to modulate the effects of her becoming vulnerable. Sophia describes her routine on the farm:

My typical days vary depending on what day it is. During the week, I usually go to my farm with my assistant (…) And, uh, then there are always seasonal tasks that come along. We now have the sheep shearing ahead of us and we have the hay work. These are the two major tasks that we have done in the last few days (…) then there is usually a short talk with, uh, my employee, my assistant and we decide what to do next.

(Interview, 14 July 2021)

Sophia’s daily life is entangled with material and nonmaterial agentic forces, including the seasonal rhythms of agricultural tasks. Her assistant plays a key role in this dynamic, and their synchronised intra‐actions are reflected in Sophia’s use of ‘we’.

The pandemic disrupted Sophia’s routines and ways of working. The cancellation of guest visits had a significant impact on her financial stability. She particularly mourned the loss of her work at a retirement home, where she had visited with her animals and built meaningful relationships. The role of her personal assistant evolved from one of support to a crucial element in helping Sophia adapt to these changes. Meanwhile, the operational rhythms of her farm were further restricted by pandemic‐related regulations, including the wearing of masks and physical distancing.

Before the pandemic, Sophia’s involvement in her local church community provided spiritual support and a social network. The shift to digital church services during the pandemic created significant barriers: ‘We had no church services. I could not yet handle computers as well as now and so we were actually completely excluded’ (interview, 14 July 2021). This exclusion became even more pronounced due to her difficulties navigating inaccessible digital platforms and engaging with governmental authorities:

I don’t know if you can imagine. But you have to listen through the homepages and there are a hundred thousand links and information. These are things where I personally think that people with disabilities are excluded because the accesses are not barrier‐free, absolutely not. And then they say just take a sighted help.

(Interview, 14 July 2021)

The pandemic‐induced digital transformation demanded significant ingenuity and adaptability from Sophia. Confronted with an inaccessible and systemically ableist framework that failed to address her specific needs, she developed strategies to advocate for her rights and secure entrepreneurial grants.

I tried to clarify many things over the phone, but most people were in home office. I always had to find out the private numbers of the employees to talk to them and say what I needed, otherwise I always just heard, please visit our homepage at www. then enjoy.

(Interview, 14 July 2021)

Her efforts culminated in a significant personal triumph:

For me personally, a sense of achievement was the first time I managed to establish a connection and participate for an hour and a half on the Internet.

(Interview, 14 July 2021)

Sophia’s agency became increasingly entangled with her mother’s deteriorating health. Concerns about her mother’s vulnerability to infection led to increased self‐isolation, which in turn reshaped her interactions with her personal assistant. Feeling compelled to limit personal contact, she chose to meet her assistant only when absolutely necessary. This recalibration culminated in a significant event when she decided to commute alone to her farm, a route that involved several train changes of varying accessibility. Although exempt from the regulations requiring face masks in public places, Sophia decided to wear one to protect her mother’s health. However, this decision inadvertently disrupted her sensory navigation, which relies heavily on acoustic and tactile feedback to compensate for her visual impairment. This challenge was magnified by the almost total absence of passers‐by who might otherwise have offered assistance or guidance. While attempting to board an express train, Sophia misjudged the gap and fell, fracturing her ankle and triggering a series of cascading effects. The absence of immediate rehabilitation services extended the duration of her movement restrictions, affected her ability to manage the farm and forced her to rely on donations to cover necessary medical treatment for her animals.

Her diary entries from September 2021 to June 2022 reveal her continued resilience and advocacy in the midst of an ongoing struggle to secure equal rights and opportunities. These entries also reflect the debilitating toll of the constant effort required to navigate inaccessible systems, including ongoing health challenges and the financial burden exacerbated by steep increases in electricity costs.

Although Sophia has had access to limited forms of support, such as pandemic‐related financial assistance, her economic stability remains precarious. Her daily life, which includes managing her farm, her health and her emotional well‐being, is intertwined with the wider socioeconomic and sociomaterial challenges of intersecting crises. The animals on her farm, essential to her livelihood, function as coagents within the material‐discursive entanglements that shape her life. Beyond emotional support, these interactions form a relational kinship, fostering a shared agency that underpins Sophia’s ability to endure and adapt in the face of financial, health and systemic adversity.

## 8 Onto‐Epistemic Boundaries of Intellectual Dis/Ability

Elisabeth, a woman in her 50s, has lived and worked in an institutional care setting for more than 2 decades. Her narrative spans three interviews and four video diary entries from summer 2021 to August 2022. Elisabeth's life prior to COVID‐19 bridged multiple domains. She navigated her roles as a resident and service user labelled with intellectual dis/ability within the institutional world while simultaneously serving as a self‐advocate for disability rights and as a knowledge producer and lecturer engaged in teaching and inclusive research at a university. These roles enabled her to act as spokesperson for other residents, especially women with dis/abilities confined to institutions. This combination provides unique insights into the ways knowledge, power and agency materialise in and outside institutional settings, shaping the conditions of existence and modes of relating.

Elisabeth's narrative was chosen because it highlights life in institutional care during the pandemic, marked by disproportionate restrictions, limited freedoms and paternalistic policies. As a self‐advocate actively engaged beyond the institution, she uniquely challenged these constraints. Her dual role—as both regulated by institutional structures and an active knowledge producer—offers a lens to examine the epistemic boundaries of how intellectual dis/ability is shaped, negotiated and resisted within material‐discursive realities.

Although her external engagements earned her recognition in academic and activist networks, they also required constant negotiation with institutional authorities, who tolerated but did not actively support her efforts:

They didn’t like it when I had appointments outside. They always asked exactly what I was doing. But I didn’t care, and eventually, they accepted it. Still, it was annoying.

(Interview, 25. August 2021)

Through her capacity as a self‐advocate, Elisabeth persistently defended her external engagements while also championing the needs of fellow residents, demonstrating how her role as a mediator between these worlds challenged institutional norms.

The onset of COVID‐19 disrupted Elisabeth's hard‐won privileges and routines, reshaping the spatial and temporal dimensions of her life. Institutional restrictions, including curfews, visitation bans and territorial limitations, were implemented abruptly and without comprehensible explanations. Elisabeth’s life, which usually included her routines in the sheltered workshop, all of a sudden felt deprived. These measures materially transformed the institution into what Elisabeth described as a ‘prison’ (interview, 25 August 2021), stripping residents of their autonomy and reducing their physical movement to their rooms, the building and occasionally the garden. The lack of intelligible communication compounded residents’ confusion and insecurity:

The question came up how long does it last ehm where can we go, can I visit someone, can I go on vacation? We didn't know what we were allowed to do and what not. Can we go shopping now or not, what is it and how contagious is it?.

(Interview, 25 August 2021)

The ability of institutional managers to respond was itself caught up in delayed and ambiguous directives from the federal government. However, they did not make use of the discretion opened up by the policy recommendations, nor did they make a sincere effort to communicate this information in a way that would alleviate the residents’ information gap.

Disproportionate strict interventions in fundamental freedoms were justified through a paternalistic discourse oscillating between protection, security and fear. This discourse targeted individuals with intellectual dis/abilities and labelled them as ‘vulnerable groups,’ incapable of comprehending the necessity for and adhering to protective measures. The de facto material confinement of residents, lasting far longer than restrictions for the general population, entangled with the silencing of their voices, reinforced essentialised images of individuals with intellectual dis/abilities as unknowledgeable, dependent and hence vulnerable. Elisabeth's narrative reveals her struggle and anger:

What are we allowed to do? We only got information when we got upset.

(Interview, 25 August 2021)

Her externally developed experiential knowledge provided her with a sense of her entitlement to rights. Unlike many other residents, who had long been conditioned to acquiescence, as the narratives from other research participants in institutional settings portray, Elisabeth resisted institutional paternalism by asserting her agency:

When I was left alone as an advocate we wrote to the authorities that we want to have the information on time.

(Interview, 25 August 2021)

The shrinking of her physical environment, including the institutional practices of keeping her uninformed, sparked her activist forces. Elisabeth mobilised internal allies, including a supportive caregiver, to navigate institutional constraints and organise measures such as indoor visitation schedules and vacation permissions for residents. Notably, Elisabeth's sense of responsibility extended to prioritising the collective well‐being of residents over her own comfort:

And I could have been exempted from the mask, but I didn’t want to, because I thought that would be unfair to the others.

(Interview, 25 August 2021)

As the pandemic progressed, the emotional toll of navigating these challenges became evident. Elisabeth's frustration with the institutional authorities' refusal to provide clarity, coupled with the isolation resulting from the absence of her external social contacts, negatively impacted her well‐being. She expressed this sense of deprivation vividly:

Yes, I missed the contact, or like saying, okay, now I’m going to a concert or like I go swimming or something, or I go to a café and even if I just sit there and drink my raspberry soda and watch people a bit.

(Interview, 25 August 2021)

The confined environment, prolonged isolation and escalating conflicts and verbal abuse among residents prompted a desire for independence:

It has come to the point that I want to move out of the institution.

(Video diary, 17 May 2022)

Yet, her aspirations to move out were met with systemic barriers and an absence of institutional support, forcing her to abandon her plans. This internal dissonance is evident in her reflections:

I’ve thought to myself: “how well off is someone who has their own apartment, even if they can’t go outside”. It’s not mentally good, because they can’t talk to anyone.

(Interview, 31 August 2022)

Elisabeth’s experiences during the pandemic illustrate how institutional practices and protective discourses, framed around notions of security and vulnerability, became mechanisms of control and exclusion. The intertwining of institutional power‐knowledge regimes and ableist discourses reinforced epistemic violence by portraying people with intellectual dis/abilities as unknowledgeable and incapable.

Living at the onto‐epistemic boundaries of intellectual dis/ability, Elisabeth navigated a complex interplay of institutional paternalism, systemic exclusion and her own relational agency. By mobilising her experiential knowledge and advocacy skills, she sought to challenge the institutional ethos and reconfigure the restricted spaces afforded to her and her peers. However, her inability to achieve her goal of independent living reveals the profound limitations imposed by structural inequalities and the pervasive culture of dependency that defines institutional care settings, offering a lens to understand the co‐construction of intellectual dis/ability within institutional environments.

## 9 Vulnerability Effects: An Emergent Framework

To understand the dynamics of vulnerability, we have leveraged the analytical affordances of agential realism. The reread narratives of John, Sophia and Elisabeth disentangle the mechanisms that create and sustain categories of vulnerability. The boundaries imposed by societal, institutional and political forces do not merely delineate who is vulnerable; they *actively shape* the sociomaterial conditions of existence for those navigating these labels.

However, these effects are not pre‐existing states or passive outcomes but *relational processes of becoming*, simultaneously produced by systemic, material and discursive conditions and generating further impacts that recursively shape these conditions. They are both *products* of specific sociomaterial arrangements and *catalysts* for subsequent effects or transformations—whether through perpetuating inequalities or fostering resistances and reconfigurations of agency. It is this dynamic interplay that we term *vulnerability effects*. Although the analysis presented here draws on only three distinct narratives, the mechanisms and dynamics of vulnerability effects resonate across broader contexts, reflecting patterns observed throughout the project. Although these findings are situated within Austria's specific policy landscape, they highlight systemic processes of vulnerability production that extend to other Global North contexts, albeit shaped by local institutional and sociopolitical conditions.

Building on Carol Thomas’s (2007, 2012) distinction between disablism and impairment effects, *vulnerability effects* parallels but also departs from her framework. Thomas describes disablism as the socially imposed restrictions and psychoemotional harms that people categorised as impaired experience, whereas impairment effects refer to the unavoidable biosocial impacts of impairments on embodied functioning, which are always culturally mediated rather than purely biological (Thomas 2012, 211). Although we align with Thomas's critique of essentialist views of disability, *vulnerability effects* rejects a rigid bifurcation between disablism and impairment effects. Instead, it situates vulnerability as an emergent phenomenon that is co‐constituted through the intra‐action of material, systemic and corporeal realities (Barad 2007).

*Vulnerability effects* integrates a dynamic view of agency, expanding beyond the psychoemotional dimensions of disablism. Although Thomas powerfully critiques the internalised oppression and relational harms of disablism, *vulnerability effects* acknowledges both the ways individuals are affected by and how they navigate and resist these conditions through creative and embodied practices. This dual framing captures the iterative and recursive nature of *vulnerability effects* as both products and catalysts, linking their emergence to broader systems of dis/ability while also recognising their transformative potential.

*Vulnerability effects* hinge between two interrelated onto‐epistemological threads that illuminate the interplay of seemingly opposing forces—activist affordances and debilitation.

Activist affordances, as conceptualised by Dokumacı (2023), refer to the micropractices of resistance and adaptation through which individuals create possibilities for existence and recognition within systems that marginalise them. These affordances foreground the embodied and relational strategies that enable individuals to navigate oppressive sociomaterial conditions.

Yet, these acts of resistance unfold within contexts where agency is continually worn down by systemic forces. Drawing on Puar's (2020) concept of debilitation, we understand this as an active process through which sociopolitical and socioeconomic systems generate vulnerability by constraining agency and reinforcing material inequalities. Debilitation not only opposes activist affordances but also interacts recursively with them, as the exertion required for resistance is met with enduring systemic barriers.

Elisabeth's experiences reveal this dynamic interplay. Her institutional confinement, shaped by systemic neglect and paternalistic norms, severely restricted her autonomy but simultaneously catalysed her advocacy efforts. Through these acts of resistance, she worked to reconfigure epistemic and spatial boundaries, challenging the very structures that excluded her from societal discourse. However, the systemic neglect she sought to contest also undermined her efforts, eroding her aspirations for independent living and diminishing her capacity for sustained resistance over time.

Sophia's narrative highlights a different dimension of this dynamic. The inaccessibility of physical and digital environments, ostensibly designed to support activity, instead reproduced exclusion and constrained her autonomy. These barriers, rooted in structural inequalities, demanded constant acts of ingenuity and advocacy, which became critical for sustaining her resilience. Her relational kinship with her animals offered a significant source of stability and agency, but the persistent need to navigate exclusionary systems exacted a cumulative toll, depleting her physical and emotional reserves.

John's experiences further illustrate the recursive interaction of debilitation and activist affordances. The compounded pressures of shifting support systems, evolving family dynamics and overlapping global crises created cycles of anxiety and destabilisation that required ever‐intensifying coping mechanisms. Although he initially developed adaptive routines to manage these challenges, the cumulative strain led to his hospitalisation, demonstrating how systemic forces can push even adaptive strategies to their breaking point. His story illuminates how shifting social dynamics—eroding solidarity and increasing hostility—destabilised his sense of belonging and magnified his vulnerability.

Framing *vulnerability effects* as recursive moves beyond static or linear understandings of vulnerability. The reciprocal relationship between debilitation and activist affordances is key to understanding how vulnerabilities are not passively endured but are actively contested and reshaped. Yet, such navigation can be taxing, as the agency exercised is often fragile (Naraian 2019). Sociopolitical fluctuations and the dwindling energy reserves people can muster impact their resilience, as do the structures and processes of institutions that often compound vulnerability. Our findings challenge the urge to merely encourage individuals to assert further agency under neoliberal paradigms. Our study points to the need for focusing on material‐discursive practices that craft *vulnerability effects*, advocating for transformative strategies to actively reform the conditions fostering them. By understanding vulnerability as both a product and a catalyst, we can interrogate the structural underpinnings of inequality while fostering pathways for transformation. This framing urges a shift in crisis response strategies from addressing immediate impacts to dismantling the systemic mechanisms of debilitation and creating conditions that sustain enduring agency.

## 10 Conclusion

As the immediacy of the pandemic fades, there is a critical risk of structural amnesia where lessons from the crisis are overshadowed by a return to entrenched practices. Revisiting the pandemic through *vulnerability effects* as a conceptual lens provides a pathway to better understand the material‐discursive practices that shape vulnerability and to imagine possibilities for transformative change grounded in equity, inclusion and shared response‐ability. Although our findings are situated within Austria's policy landscape and reflect systemic patterns in the Global North, further research is needed to explore how these dynamics manifest in diverse sociopolitical settings.

Within our project, an initial prototype for inclusive crisis monitoring was implemented through the participatory *C_all Voices* project. This initiative brought together various stakeholders and supported the cocreation of arts‐based performances developed by individuals with dis/abilities, reflecting their pandemic experiences. These events not only facilitated a shared exploration of the pandemic's effects but also informed actionable recommendations for inclusive crisis management strategies. A forthcoming paper will further explore the epistemic injustices of knowledge production and participation during the COVID‐19 pandemic, offering insights into the systemic transformations required for future crises.

## Author Contributions

**Oliver Koenig:** conceptualisation (lead), data curation (supporting), formal analysis (equal), funding acquisition (lead), investigation (supporting), methodology (equal), project administration (supporting), writing – original draft (equal), writing – review and editing (lead). **Sabine Mandl:** conceptualisation (supporting), data curation (lead), formal analysis (equal), funding acquisition (supporting), investigation (lead), methodology (equal), project administration (lead), writing – original draft (equal), writing – review and editing (supporting). **Simon Reisenbauer:** conceptualisation (supporting), data curation (supporting), formal analysis (equal), investigation (supporting), methodology (equal), project administration (supporting), writing – original draft (equal), writing – review and editing (supporting).

## Ethics Statement

The research project underwent a rigorous international assessment process and received funding from the Austrian Science Fund, adhering to the applicable Austrian laws on research ethics. Given the nonclinical nature of this project, which does not involve data collection from clinical settings, formal ethical approval was not required. This compliance with ethical standards was acknowledged in the ethics statement submitted with the approved project proposal, which is reproduced here.

Cov_enable involves a range of ethical issues that will be handled with high diligence by all members of the research team. The project will follow ethical guidelines for establishing a multistage informed consent procedure for all research participants. This means that self‐consent, as well as proxy consent in the case of minors, will be sought in order to safeguard them from any harm that might arise. Additionally, we will pay particular attention to provide accessible information on the specific elements of the research design (longitudinal design, production and use of video material, and accompanying interviews). Because video data are not just used for elicitation purposes and because, through a grounded theory‐informed iterative design, new themes will evolve in the course of the study, the process of informed consent will be updated regularly, giving each study participant the possibility to withdraw their consent at any point. Participants will also be able to give only partial consent, especially concerning the scope of use of video material and images produced and edited (Wiles et al. 2008). Especially, the use of video and other visual material involves further ethical issues in relation to questions of confidentiality and representation. Here, Anderson and Muñoz Proto (2016) differentiate between ethical requirements and ethical responsibilities. Ethical requirements, among others, refer to adherence to legal regulations, in our case the European General Data Protection Regulation (GDPR). Further legal issues, especially regarding the participation of children or other vulnerable groups, concern potentially sensitive issues, such as participants disclosing current experiences of violence, harm or neglect. In such cases, we will seek to cooperate with established violence protection schemes for children, as well as ombudsperson services for people living in institutions. Ethical responsibilities refer to ethical obligations that result from epistemological orientations of the researcher(s), as well as the normative base of one's research methodologies. Thus, in a participatory approach, questions of voice, self‐determination and self‐representation must be vigorously upheld. As such, for groups perceived as being vulnerable and whose voices and (self‐directed) images are often silenced or blinded, especially video data pose a significant opportunity to shift power balances towards an emancipatory ethics of (self‐)representation (Bates 2020). Hereby, taking an ethical stance also involves the transparent disclosure and negotiation of potential dilemmas together with research participants to neither fall into the trap of paternalism on one side nor neglect the protection responsibility on the other side, for example, in cases when research participants want to (visually) disclose experiences of violence. Whenever possible, we seek to navigate such potential dilemmas through technological means, such as using filters (e.g., black and white, aquarelle) or pixelation for videos and pictures.

The use of digital technologies and visual representations also poses questions of data security and privacy that have so far not been widely addressed in the context of remote data collection, especially in the context of participatory research approaches. It is important to consider that security embraces usability to avoid additional barriers in technology usage for disabled people. The aim is to ensure accessibility and keep data secure and protected from misuse. The external advisory panel will therefore also include an expert on data security and sustainable data management. The project aims to add to the body of knowledge in this context.

Cov_enable will ensure that data will be safely stored, processed and transferred. All personal data (age, gender, year of migration, job etc.) of research participants will be stored separately and safely (on password‐secured university‐based computers) and will not be shared with third parties. All project partners will commit to these policies in a confidentiality agreement. The privacy and confidentiality of the project will be assured in the phase of processing data, for example, during data analysis. The collected data will be stored for 5 years after the completion of the project at a secure data storage port such as Phaidra.

## Consent

The authors have nothing to report.

## Conflicts of Interest

The authors declare no conflicts of interest.

## Permission to Reproduce Material From Other Sources

The authors have nothing to report.

## Data Availability

The qualitative data supporting this article, including personal audio diaries, written diaries and individual interviews, are securely stored in compliance with the European General Data Protection Regulation (GDPR) to safeguard sensitive information. Access to this data is restricted to members of the research team who have been explicitly recognised by the research participants as per the conditions stipulated in their written consent. Therefore, the data are not publicly available to ensure privacy and confidentiality.

## Endnotes

^1^
We understand dis/ability as a continuum of difference among many in the complex human formation that shows that perceptions of normalcy are always subject to interpretation/perspective and context and affected by relational, political and material‐discursive practices and forces. The slash indicated by dis/ability signifies this “interconnectedness, transcending separate states and underscoring their reliance on embodied experience, materiality and discourse” (Bozalek and Fullagar 2021, 3).

^2^
Although our research engages with participant narratives, we do not approach them as isolated, human‐centred accounts of subjective experience. Rather, we understand narratives as emergent configurations shaped through entangled intra‐actions of material, institutional and discursive forces within the research process.
